# Hypervirulent *Klebsiella pneumoniae* (hypermucoviscous and aerobactin positive) infection over 6 years in the elderly in China: antimicrobial resistance patterns, molecular epidemiology and risk factor

**DOI:** 10.1186/s12941-018-0302-9

**Published:** 2019-01-21

**Authors:** Chao Liu, Jun Guo

**Affiliations:** 1 Department of Respiratory Medicine, Peking Union Medical College, Chinese Academy of Medical Sciences, Beijing, China; 2 Department of Respiratory Medicine, Beijing Tsinghua Changgung Hospital, School of Clinical Medicine, Tsinghua University, No. 168 Litang Road, Changping District, Beijing, 102218 China; 3 Department of Geriatric Respiratory Medicine, Chinese PLA General Hospital, Beijing, China

**Keywords:** *Klebsiella pneumoniae*, Hypervirulent, Hypermucoviscous, Aerobactin, The elderly, Risk factor

## Abstract

**Background:**

The definition of hypervirulent *Klebsiella pneumoniae* (hvKp), traditionally regarded as hypermucoviscosity, is controversial. However, data based on both phenotype (hypermucoviscous) and genetic (aerobactin) criteria are limited.

**Methods:**

A retrospective study was conducted in 175 geriatric patients between January 2008 and January 2014. The clinical and molecular data, including antimicrobial susceptibility testing, extended-spectrum-β-lactamase (ESBL) production, virulence gene, and multilocus sequence typing of the hvKp-group (hypermucoviscosity and aerobactin positive) were compared with those of classic *K. pneumoniae* (cKp) isolates.

**Results:**

Of 175 Kp isolates, 45.7% were hvKp. In pathogenicity, *K1*, *K2*, *magA*, *rmpA*, and *rmpA2* genes were strongly associated with hvKp (P < 0.01). In the hvKp group, invasive infections (P < 0.000), liver abscess (P = 0.008), abdominal infection (P = 0.002) and septic shock (P = 0.035) are significantly higher than cKp group. Patients with better nutritional status were frequently infected with hvKp. However, host inflammatory reaction is most severe in hvKp group. Patients with diabetes (odds ratio [OR] = 2.548) and digestive diseases (OR = 2.196) are more likely to be infected with hvKp. Importantly, the detection of hvKp isolates increased from January 2008 to January 2010, January 2010 to January 2012, and January 2010 to January 2014 (12, 30, and 48 isolates, respectively). Overall, 16.3% of hvKp isolates produced ESBLs and 20.0% were MDR-hvKp. Multivariate analysis implied that infection occurred in the ICU (OR = 5.826) and patients with indwelling stomach tubes (OR = 6.461) are independent risk factors for ESBL-hvKp infection.

**Conclusions:**

HvKp, especially ESBL-hvKp and MDR-hvKp, is emerging in the elderly. It is essential to enhance clinical awareness and management of hvKp infections.

**Electronic supplementary material:**

The online version of this article (10.1186/s12941-018-0302-9) contains supplementary material, which is available to authorized users.

## Introduction

*Klebsiella pneumoniae* (Kp) are Gram-negative bacteria that can cause various infections. There are mainly two pathotypes that pose a threat to our health: hypervirulent (hvKp) and classical (cKp). The most common subtype of the *K. pneumoniae* strains is classic *K. pneumoniae* (cKp) notorious for their resistance to common antibiotics [1–3]. An emerging subtype, termed hypervirulent *K. pneumoniae* (hvKp), was first described in 1986 [4]. The hvKp strains exhibit unique features compared to cKp. The hvKp strains exhibit hypermucoviscosity to cause various severe infections in immunocompetent and young healthy individuals in addition to diseased patients [5–9], liking pyogenic liver abscesses (PLA) [4, 10]. However, the definition of hvKp is controversial. Host, pathogen, and host–pathogen interactions should be considered comprehensively for defining hvKp. However, most published studies have focused on the bacteria alone. A previous study concluded that major histocompatibility complex (MHC) variants, eating habits, nutritional status, and gut microbiota composition are essential host factors to investigate to enhance our understanding of the hypervirulence phenomenon [11]. Moreover, some controversies exist about the relationship between the virulent and morphological phenotype (hypermucoviscosity) [12, 13]. Using in vitro and in vivo assays, various studies showed that few hypermucoviscous *K. pneumonia* (hmvKp) strains are associated with high virulence [12, 13]. In animal models, hypermucoviscous *K. pneumonia* did not cause more severe infections and a higher mortality rate than non-hypermucoviscous *K. pneumonia*. In vitro and in vivo experiments showed that a few (1/5) hypermucoviscous *K. pneumoniae* isolates had a high virulence. Thus, identifying hvKp by the string test alone is not sufficient [11, 14].

Recently, aerobactin has been regarded as a critical virulence factor for hvKp [14–16], which is often concomitant with the mucoid phenotype. Based on this finding, a multi-centre research in China first stated the clinical and molecular characteristics of hvKp (defined as aerobactin-positive) isolate [14]. The results showed that invasive infections (especially PLA), hypermucoviscosity and most of virulence factors (*K1*, *K2*, *K20*, *rmpA*) genes are highly associated with aerobactin-positive Kp. In addition, some studies have reported that iron acquisition factors and the genes encoding the hypermucoviscous phenotype are located on the same virulence plasmid, which is not frequently present in cKp strains [5, 17–19]. Therefore, aerobactin combined with hypermucoviscosity may be a defining hvKp trait. Additionally, the elderly often has various underlying diseases, poor nutritional status and atypical manifestations.

To date, no data about antimicrobial susceptibility, epidemiology and risk factor of hvKp in the elderly has been described. Thus, we conducted a comparison of hvKp (hypermucoviscous- and aerobactin-positive) and cKp considering the host nutritional status, pathogen and host–pathogen interactions.

## Methods

### Patients

A retrospective study was conducted on *K. pneumoniae* culture-positive patients diagnosed at Chinese PLA General Hospital between January 2008 and January 2014. Duplicate isolates from the same patient were excluded. The basic demographics and clinical characteristics (underlying diseases, invasive procedures, nutritional status, and survival) of patients infected by *K. pneumoniae* were collected. Sequential Organ Failure Assessment (SOFA) scores were evaluated within the first 24 h after admission. To further assess the host response and nutritional status between the two pathotypes, we monitored white blood cell count (WBC), percentage of neutrophils (NEU%), total protein (TP) and albumin (ALB) as biomarkers. The study was approved by the Chinese PLA General Hospital Ethics Committee and the Guidelines for Human Experimentation (PR. China) were followed throughout. The main inclusion criteria were (1) the definition of the elderly has being 65 years old or older (≥ 65 year); (2) at least one *K. pneumoniae* positive culture; (3) Patients with all the indicators(WBC, NEU %, TP, ALB, SOFA score) were recruited in this study when their clinical specimens were identified as Kp. The exclusion criterions were (1) insufficient clinical data (lacking one of these above indicators) or bacterial strain sample storage and (2) co-infection cases. Infections were considered to be community-acquired infections if *K. pneumoniae*-positive culture was obtained from a sample isolated upon admission to the study center within 24 h. Cases without these conditions were defined as nosocomial infections.

### Clinical *K. pneumonia* isolates

These specimens were from sputum, urine, blood and drainage fluid. The standardized isolation, culture and identification were conducted in the Department of Clinical Microbiology. All strains were stored at − 80 °C. All the strains were identified by the API 20 NE system and the Vitek II system. Moreover, species identification was further confirmed by 16S rRNA gene sequencing. The definition of hvKp required that both hypermucoviscosity and aerobactin were positive. Hypermucoviscosity was confirmed by the positive string test as previously described [20].

### Antimicrobial susceptibility testing and phenotypic confirmation of extended spectrum beta lactamases (ESBL)

Antimicrobial susceptibility testing was conducted using the microbroth dilution method as previously described [6]. The following antibiotic agents were included: Amikacin, Gentamicin, Ampicillin/Sulbactam, Aztreonam, Cefazolin, Cefepime, Ceftriaxone, Ceftazidime, Ciprofloxacin, Levofloxacin, Piperacillin/Tazobactam, Trimethoprim/Sulfamethoxazole, Imipenem, Meropenem and Tobramycin. The results were interpreted using the 2017 Clinical and Laboratory Standards Institute (CLSI) guidelines. ESBL was confirmed by agar dilution test using ceftazidime and cefotaxime combined with clavulanate [14]. Multidrug-resistant isolate was defined as resistant to three or more antimicrobial classes [21].

### Detection of virulence-associated gene and capsular serotype-specific (*cps*) genes

Genomic DNA was extracted from all *K. pneumoniae* isolates. Polymerase Chain Reaction (PCR) for virulence-associated genes (such as *rmpA, rmpA2, magA* and *aerobactin*) were conducted as previously described [14, 22, 23]. Capsular serotype-specific genes (*K1*, *K2*, *K5*, *K20*, *K54*, and *K57*) were amplified by PCR [14, 24]. The primers used are listed in Additional file 1: Table S1.

### Multilocus sequence typing

The primers and reaction conditions of seven housekeeping genes (*gapA*, *mdh*, *phoE*, *tonB*, *infB*, *pgi*, and *rpoB*) were utilized according to the *K. pneumoniae* MLST website (http://bigsdb.pasteur.fr.html) (Additional file 1: Table S1). Allelic profiling and sequence types (STs) determination were also confirmed using the above website. In addition, for further analyses the relationship among different STs, phylogenetic analysis of housekeeping genes was performed. The concatenation of the seven housekeeping genes of *K. pneumonia* was conducted. A dendrogram was constructed from the concatenated sequences using the neighbour-joining method (MEGA 6.05).

### Statistical analysis

SPSS software (version 20.0) was used for data analysis. Measurement data were reported as the mean ± standard deviation (SD), and count data were analysed as percentages. Student’s *t*-tests and the Wilcoxon rank-sum tests were performed for the analysis of continuous variables. The χ^2^ or Fisher’s exact test was used for categorical variables. All tests were 2-tailed. The P-value < 0.05 was considered statistically significant. To determine the risk factors for hvKp, univariate logistic regression analyses were performed. All variables with a P value < 0.05 were included in the multivariate model.

## Results

### Patient Characteristics

Between January 2008 and January 2014, 175 cases are appropriate for this study. Aerobactin-positive and hypermucoviscous strains were defined as hvKp, which was determined by PCR and string test. Eighty of 175 (45.7%) isolates were hvKp. The distribution of the main infection types in the hospital was hospital acquired pneumonia (130, 72.3%), urinary infection (28, 16.0%), abdominal infection (24, 13.7%) and bacteraemia (9, 5.14%). Overall, 170 (97.1%) patients were males and five (2.9%) were females; the mean age was 84.84 ± 8.48 years.

### Clinical characteristics (including host response and nutritional status) of hvKp infection

The basic clinical characteristics, host response and nutritional status of patients with hvKp infections are shown in Table 1. The mean age of patients infected with hvKp is significantly younger than the cKp group (83.2 ± 8.75 years vs 86.2 ± 8.04 years, P = 0.020). A significantly higher number of patients with hvKp had diabetes (76.3% versus 54.7%; P = 0.003) as their underlying diseases. Compared with the cKp group, more patients with hvKp infections presented with invasive infections (28.8% versus 6.3%; P = 0.000), liver abscess (10.0% vs 1.1%; P = 0.008), other abscesses (16.3% vs 3.2%; P = 0.035), sepsis shock (11.3% versus 3.2%; P = 0.035) and abdominal infection (22.5% vs 6.3%; P = 0.035). However, the rate of urinary infection in the hvKp group is lower (10.0% vs 21.1%, P = 0.047). In addition, stomach tube is also less common in the hvKp group (56.3% vs 74.7%, P = 0.01). With regard to the host response, both WBC (12.87 ± 4.24 vs 10.34 ± 2.95, P = 0.000) and NEU % (78.87 ± 7.60 vs 74.23 ± 7.83, P = 0.000) are higher in patients with hvKp than the cKp group. However, patients infected with hvKp are more likely to have a lower TP (65.14 ± 4.93 vs 62.96 ± 4.71, P = 0.003) and ALB (35.54 ± 2.75 vs 34.45 ± 3.44, P = 0.021). It was also noted that although the SOFA score in the hvKp group is higher (6.84 ± 2.81 vs 4.93 ± 2.59, P = 0.000), the mortality at 28 days (17.5% vs 17.9%, P = 0.946) was not significantly different between the two groups (Table 1).

**Table 1:** Clinical and microbiological characteristics, host response and nutritional status of hvKp

Characteristic	HvKp (80)	cKp (95)	P value
K serotype			
*K1*	26 (32.5%)	3 (3.2%)	0.000
*K2*	11 (13.8%)	1 (1.1%)	0.001
*K5*	1 (1.3%)	0 (0%)	0.276
*K20*	2 (2.5%)	5 (5.3%)	0.354
*K54*	2 (2.5%)	3 (3.2%)	0.795
*K57*	6 (7.5%)	7 (7.4%)	0.974
*rmpA*	65 (81.3%)	17 (17.9%)	0.000
*rmpA2*	58 (72.5%)	19 (20.0%)	0.000
*magA*	63 (78.8%)	58 (61.1%)	0.012
Basic demographics			
Age	83.2 ± 8.75	86.2 ± 8.04	0.020
Male	77 (96.3%)	92 (96.8%)	0.837
Underlying diseases			
Pulmonary disease	73 (91.3%)	90 (94.7%)	0.363
Diabetes	61 (76.3%)	52 (54.7%)	0.003
Cardiovascular disease	40 (50.0%)	58 (61.1%)	0.142
Cerebrovascular disease	9 (11.3%)	20 (21.1%)	0.082
Cancer	21 (26.3%)	28 (29.5%)	0.636
Surgery within 1 mo	6 (7.5%)	11 (11.6%)	0.364
Digestive disease	25 (31.3%)	20 (21.1%)	0.124
Catheter			
Central intravenous catheter	50 (62.5%)	65 (68.4%)	0.411
Urinary catheter	57 (71.3%)	79 (83.2%)	0.059
Tracheal catheter	24 (30.0%)	33 (34.7%)	0.505
Stomach tube	45 (56.3%)	71 (74.7%)	0.01
Drainage tube	4 (5.0%)	1 (1.1%)	0.119
Infection type			
HAP	62 (77.5%)	68 (71.6%)	0.372
Urinary infection	8 (10.0%)	20 (21.1%)	0.047
Invasive infection	23 (28.8%)	6 (6.3%)	0.000
Bacteraemia	5 (6.3%)	4 (4.2%)	0.543
Liver abscess	8 (10.0%)	1 (1.1%)	0.008
Other abscess	13 (16.3%)	3 (3.2%)	0.003
Abdominal infection	18 (22.5%)	6 (6.3%)	0.002
Sepsis	41 (51.3%)	40 (42.1%)	0.227
Septic shock	9 (11.3%)	3 (3.2%)	0.035
Host response			
WBC	12.87 ± 4.24	10.34 ± 2.95	0.000
NEU%	78.87 ± 7.60	74.23 ± 7.83	0.000
Nutrition status			
TP	65.14 ± 4.93	62.96 ± 4.71	0.003
ALB	35.54 ± 2.75	34.45 ± 3.44	0.021
SOFA score	6.84 ± 2.81	4.93 ± 2.59	0.000
Infection occurred in ICU	13 (16.3%)	14 (14.7%)	0.783
Relapse	5 (6.3%)	5 (5.3%)	0.779
Mortality at 28 days	14 (17.5%)	17 (17.9%)	0.946

Underline values indicate statistical significance

*TP* total protein, *ALB* albumin; *HAP* hospital acquired pneumonia, *WBC* white blood cell count, *ESBLs* extended spectrum beta lactamases, *NEU%* percentage of neutrophils

### Genetic characteristics of hvKp vs cKp

Previous reports showed that the virulence-associated genes *rmpA*, *rmpA2*, *magA* and (*K1*, *K2*, *K5*, *K20*, *K54*, *and K57*) genes for capsular K antigens are associated with hvKp [25–27]. All isolated strains were tested for the above genes by PCR. *K1*, *K2*, *rmpA*, *rmpA2* and *magA* were highly associated with hvKp (P = 0.000, 0.001, 0.000, 0.000, and 0.012, respectively). However, *K5*, *K20*, *K54*, and *K57* were not associated with hvKp (P = 0.276, 0.354, 0.795, and 0.974, respectively). There is no strain in cKp group with K5 (Table 1).

### Antimicrobial resistance and prevalence of ESBL genes among *K. pneumoniae* isolates

The resistance rate of almost all antibiotic agents for cKp was significantly higher than that of the hvKp group, with the exception of ampicillin, imipenem, and meropenem (Additional file 1: Table S2). All hvKp strains were resistant to ampicillin. Two hvKp isolates were resistant to carbapenems. Among hvKp strains, 16 strains (20.0%) were identified as multi-drug resistant bacteria (MDR). Fifty-one strains were identified as ESBL-producing, which was more common in the cKp group (40.0% vs 16.3%, P = 0.001). In the hvKp group, 16.3% (13/80) samples were ESBL-producing isolates, and 2 of them presented with carbapenems resistance. The detailed information about the 13 ESBL-producing hvKp strains is shown in Table 2.

**Table 2:** Clinical and microbiological characteristics of ESBL-producing hvKp isolates

Clinical characteristic	P14	P32	P34	P45	P51	P65	P92	P133	P145	P212	P221	P233	P237
Age		86	73	89	90	94	79	85	86	93	91	93	86
Gender	M	M	M	M	M	M	M	M	M	M	M	M	M
Clinical department	Cardiology	ICU	Urology	CCU	Respiratory	ICU	Endocrinology	Respiratory	Gastroenterology	ICU	Cardiology	Respiratory	CCU
Date of specimen (yr/mo/day)	2011/04/18	2010/10/10	2010/07/14	2008/10/24	2008/07/22	2010/10/14	2011/08/11	2011/07/11	2013/05/19	2014/01/21	2013/05/15	2013/09/11	2013/01/16
Main underlying diseases	Cardiovascular diseases	UIP	Prostate Disease	CHD	Bronchiectasis	Diabetes	Diabetes	UIP	Diabetes	UIP	Heart failure	Diabetes	Diabetes
Tube	CVC; ureter; stomach tube	CVC; ureter; stomach tube; tracheal catheter	CVC; ureter; stomach tube; tracheal catheter	CVC; ureter; stomach tube	Ureter; stomach tube	CVC; ureter; stomach tube; Tracheal catheter	CVC; ureter; stomach tube; tracheal catheter	Non	Non	CVC; ureter; stomach tube; tracheal catheter	CVC; ureter; stomach tube	Stomach tube	Stomach tube
Specimen type	Sputum	Sputum	Urine	Urine	Sputum	Sputum	Sputum	Sputum	Sputum	Sputum + blood	Urine	Sputum	Sputum
Infection type	Pneumonia	Sepsis	Sepsis	Urinary infection	Pneumonia	Sepsis	Sepsis	Pneumonia	Pneumonia	Sepsis shock	Urinary infection	Pneumonia	Pneumonia
WBC (10^9^/L)	14.36	11.35	13.14	7.33	8.34	7.38	13.2	8.47	12.26	14.1	8.3	9.45	13.3
NEU (%)	82.9	87.6	81.3	69.2	67.5	66.3	83.1	70.5	78.3	64.4	69.3	81.3	76.3
TP (g/L)	67	63.7	75	61	58	60	61	69	68	61	64	62	67
ALB (g/L)	35.2	30.7	35.7	34.5	31.3	31.5	32.9	39	36.9	32.6	37.2	36.5	37.8
MDR	Y	Y	Y	Y	Y	Y	Y	N	N	Y	Y	Y	N
Antibiotic resistance type	Penicillins; cephalosporins; aminoglycosides; beta-lactamase inhibitor; quinolones	Penicillins; cephalosporins; aminoglycosides	Penicillins; cephalosporins; sulfonamides	Penicillins; cephalosporins; aminoglycosides	Penicillins; cephalosporins; aminoglycosides; beta-lactamase inhibitor; quinolones; Sulfonamides	Penicillins; cephalosporins; aminoglycosides; beta-lactamase inhibitor; quinolones; Sulfonamides	Penicillins; cephalosporins; quinolones	Penicillins; cephalosporins;	Penicillins; cephalosporins	Penicillins; cephalosporins; aminoglycosides	Penicillins; cephalosporins; aminoglycosides	Penicillins; cephalosporins; sulfonamides	Penicillins; cephalosporins
Empiric Therapy	CIP + CAZ	MEM + ISE	CMZ	IPM	MXF	CIP + CAZ	IPM + ISE	MXF	MXF	TZP + ISE	MXF	CIP + CAZ	CIP + CAZ
Switched Therapy	MEM	MEM	MXF	TZP	MEM	CAZ + TZP	IPM	MXF	MXF	MEM + CIP	MXF	CIP + ISE	CIP + ISE
SOFA score	6	7	10	3	5	7	8	3	5	11	5	7	6
Clinical outcome	Survived	Survived	Survived	Survived	Survived	Survived	Survived	Survived	Survived	Died	Survived	Survived	Survived
String test length (mm)	100	30	50	100	40	20	200	45	20	60	8	8	50
Virulence-associated genes													
*rmpA*	−	−	−	+	+	+	+	−	+	+	+	−	+
*rmpA2*	−	−	−	+	+	+	+	−	+	+	−	−	+
*magA*	+	+	+	+	+	+	+	+	+	+	−	+	+
*aerobactin*	+	+	+	+	+	+	+	+	+	+	+	+	+
*cps* genes													
*K1*	−	−	−	−	−	−	+	−	−	−	−	−	+
*K2*	−	−	−	−	−	−	−	−	−	−	−	−	−
*K5*	−	−	−	−	−	−	−	−	−	−	−	−	−
*K20*	−	−	−	−	−	−	−	−	−	−	−	−	−
*K54*	−	−	−	−	−	−	−	−	−	−	−	−	−
*K57*	−	−	−	−	−	−	−	−	−	+	−	−	−
MLST genotyping	2899	2892	34	2888	1264	412	2898	2920	23	17	2836	101	23
Clone complex	Singleton	CC292	CC34	CC1	CC11	CC412	CC1	singleton	CC23	CC17	CC292	CC101	CC23

*M* male, *ICU* intensive care unit, *CCU* coronary care unit, *UIP* usual interstitial pneumonia, *CHD* coronary heart disease, *CVC* central venous catheter, *CIP* ciprofloxacin, *MEM* meropenem, *IPM* imipenem, *TZP* piperacillin tazobactam, *ISE* isepamicin, *CMZ* cefmetazole, *MXF* moxifloxacin, *CAZ* ceftazidime, *Y* yes, *N* no

**Fig. 1:**
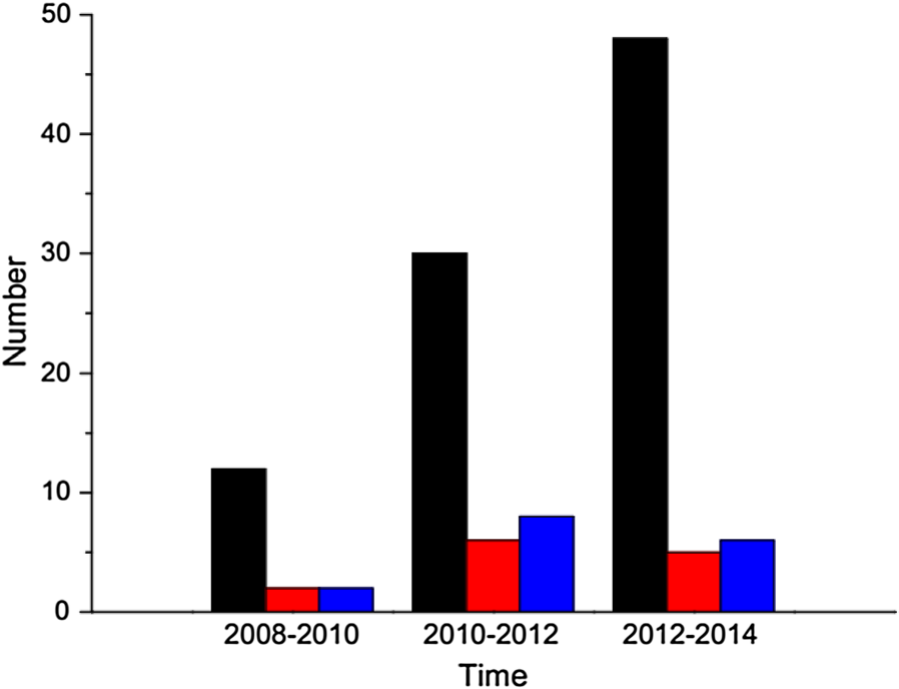
Number of hypervirulent *Klebsiella pneumoniae* (hvKp), ESBL-hvKp and MDR-hvKp strains detected between January 2008 and January 2014. (black, hvKp; red, ESBL-hvKp; blue, MDR-hvKp)

The distribution time and the rate of multi-drug resistance of hvKp were investigated. During the periods from January 2008 to January 2010, February 2010 to January 2012, February 2012 to January 2014, 12, 30, and 48 hvKp isolates were detected, respectively. At the three time points, 2, 6, and 5 ESBL-hvKp strains and 2, 8, and 6 MDR-hvKp strains were detected, respectively. Furthermore, an increase in the number of ESBL-hvKp isolates was detected during the periods from January 2008 to January 2010 (n = 2), February 2010 to January 2012 (n = 6), and February 2012 to January 2014 (n = 5). Additionally, 2, 8 and 6 MDR-hvKp stains were observed in the above three time points, respectively (Fig. 1).

### Risk factors: hvKp vs cKp

In this study, univariate regression analysis showed that diabetes (odds ratio [OR] = 2.655) and digestive diseases (OR = 2.152) were statistically significant risk factors associated with hvKp infections (Table 2). Indwelling stomach tube (OR = 0.435) is a protective factor for hvKp infection. Moreover, multivariate analysis revealed that diabetes (OR = 2.548) and digestive diseases (OR = 2.196) were independent risk factors for hvKp infections (Table 3).

**Table 3:** Risk factor for hvKp vs cKp

Variable	Univariate OR (95% CI)	P value	Multivariate OR (95% CI)	P value
Infection occurred in ICU	1.123 (0.494–2.552)	0.783		
Pulmonary diseases	0.579 (0.177–1.901)	0.368		
Diabetes	*2.655 (1.380–5.108)*	*0.003*	*2.548 (1.288–5.042)*	*0.007*
Cardiovascular disease	0.638 (0.349–1.164)	0.143		
Cerebrovascular disease	0.475 (0.203–1.113)	0.087		
Cancer	0.852 (0.438–1.657)	0.636		
Surgery within 1 mo	0.619 (0.218–1.756)	0.368		
Digestive diseases	*2.152 (1.033–4.483)*	*0.041*	*2.196 (1.003–4.806)*	*0.049*
Central intravenous catheter	0.769 (0.411–1.439)	0.411		
Urinary catheter	0.502 (0.244–1.035)	0.062		
Tracheal catheter	0.805 (0.425–1.524)	0.506		
Stomach tube	0.435 (0.229–0.824)	0.011		

Italic values indicate statistical significance

### Risk factors: ESBL-hvKp vs Non-ESBL-hvKp

Patients infected in the ICU department (OR = 5.826) and indwelling stomach tube (OR = 6.421) are significant independent risk factors for ESBL-producing hvKp infections by regression analysis (Table 4).

**Table 4:** Risk factor for ESBL-hvKp vs Non-ESBL-hvKp

Variable	Univariate OR (95% CI)	P value	Multivariate OR (95% CI)	P value
Infection occurred in ICU	*4.609 (1.208–17.591)*	*0.025*	*5.826 (1.334–25.446)*	*0.019*
Stomach tube	*5.338 (1.099–25.941)*	*0.038*	*6.461 (1.218–34.259)*	*0.028*
Relapse	3.879 (0.580*–*25.936)	0.162		
Pulmonary diseases	1.180 (0.130*–*10.713)	0.883		
Diabetes	1.046 (0.256*–*4.271)	0.950		
Cardiovascular disease	2.613 (0.139*–*9.322)	0.732		
Cerebrovascular disease	1.558 (0.285*–*8.513)	0.609		
Cancer	0.196 (0.024*–*1.609)	0.129		
Digestive diseases	0.705 (0.175*–*2.837)	0.623		
Central intravenous catheter	0.952 (0.281*–*3.233)	0.938		
Urinary catheter	0.891 (0.245*–*3.242)	0.861		
Tracheal catheter	1.579 (0.458–5.441)	0.469		

Italic values indicate statistical significance

### MLST genotypic analysis

**Fig. 2:**
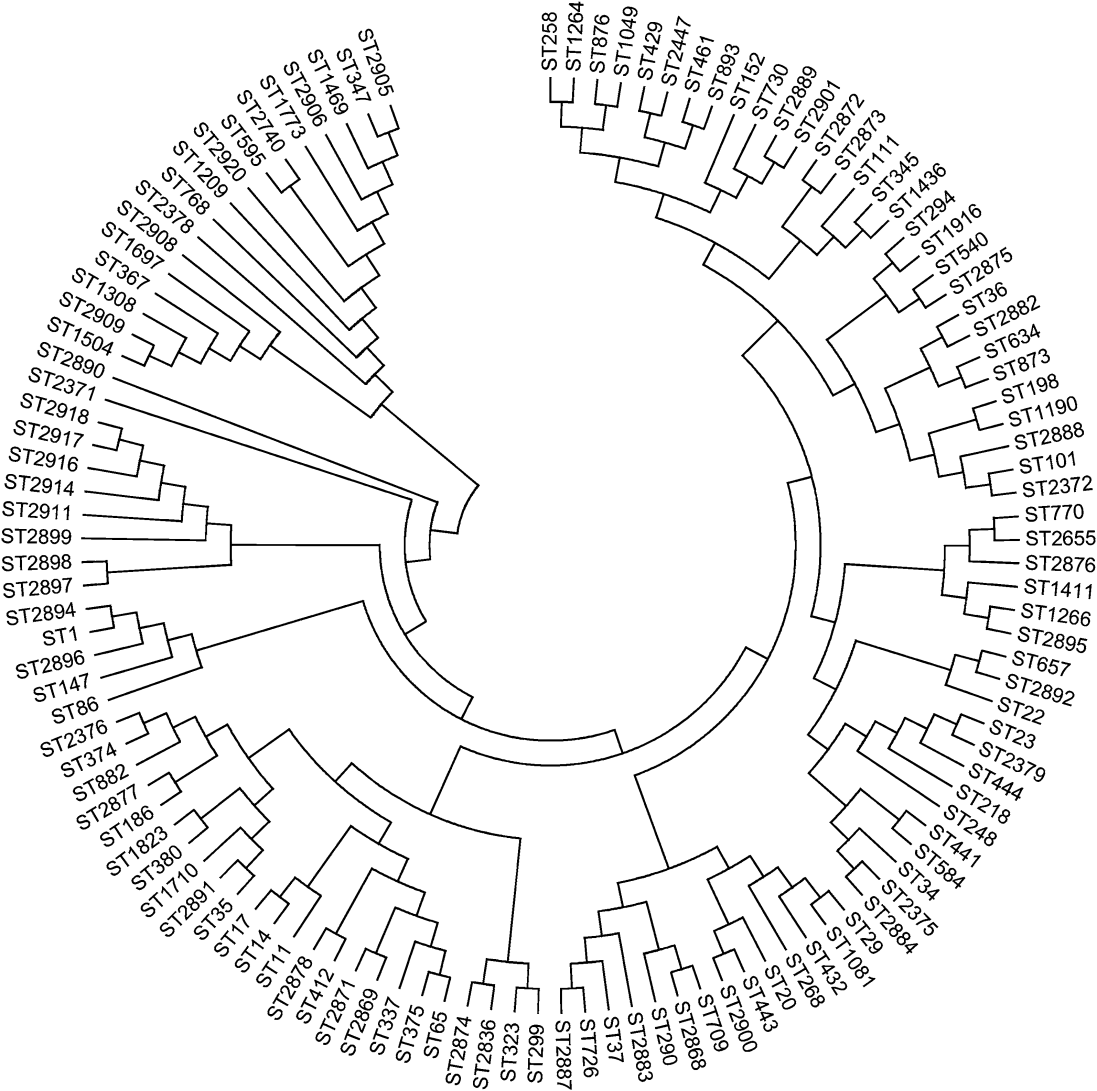
Neighbour-joining dendrogram of concatenated sequences of seven housekeeping genes from the MLST database

Among the 175 *K. pneumoniae* isolates, 119 STs were identified by MLST analysis, including 37 novel STs (ST2868–2869, ST2871–2878, ST2882–2884, ST2887–2892, ST2894–2901, ST2905–2906, ST2908–2909, ST2911, ST2914, ST2916–2918, ST2920). The most prevalent ST in this study was ST23 (n = 22;18.5%), followed by ST37 (n = 6;5.0%), ST11 (n = 5;4.2%), and ST412 (n = 5;4.2%). These 4 STs accounted for 27.7% (33/119) of the total strains. Moreover, 97 isolates identified another 97 distinct STs. ST23, ST412, ST218, ST375, and ST65 were strongly associated with hvKp, while ST11, ST37, and ST461 were more common in the cKp group. The most common clone complex (CC) of the ESBL-hvKp strains were CC1 (N = 2), CC23 (N = 2) and C292 (N = 2), followed by CC412, CC101, CC17, CC34, CC11 and two singletons. The phylogenetic tree showed that the ST347 isolate produced a serious infection (SOFA = 8), and the other STs (ST595, ST2906, ST1469) resulted in death (Fig. 2).

## Discussion

To our knowledge, our study is the first systematic study of hvKp defined as hypermucoviscosity and aerobactin positive and provides a comprehensive assessment of this definition regarding the host nutritional status, pathogen and host–pathogen interactions in the elderly. In the present study, nearly half of *K. pneumonia* (45.7%) accounted for infection in the elderly. Additionally, it is noted that, in the elderly, the detection of hvKp among the *K. pneumoniae* isolates increased from 2008 to 2014, indicating an elevated risk for hvKp infection, which is consisted with a previous study focusing on adults in China [20]. In our study, 45.7% of *K. pneumonia* were identified as hypermucoviscous through a positive string test, which is higher than a previous retrospective study conducted at a single centre in China, with a prevalence of 33% in Beijing [20]. HvKp is emerging in the elderly and may be a potential “superbug” for further clinical practice. However, the hypermucoviscous phenotype may not the unique key trait of hvKp. Moreover, patients with WBC, NEU%, TB, ALB can be included into this study. Therefore, the prevalence of hvKp in the elderly may be incorrectly estimated due to the lack of objective diagnostic methods and small sample size.

The resistance rate to common antibiotics (except carbapenems) in hvKp strains was still significantly lower than that in the cKp group in this study, particularly with regard to ESBLs. In addition, 16.3% of ESBL production was found among hvKp strains in our study, which is higher than previous article [14]. It is widely recognized that carbapenemase-producing hvKp (CR-hvKp) strains have cause various fatal infections, especially an outbreak in critical patients [17, 28, 29]. It was confirmed that the carbapenemase-producing plasmid could be successfully transferred into hvKp strains, leading to a large burden of disease for the public health [30]. In this study, MDR-hvKp is increasing and 2 hvKp isolates show high resistant to carbapenems in the elderly. It is alarming that CR-hvKp isolates are emerging, and it is a big challenge for medical workers to put forward new clinical intervention and prevention. Taken together, these data revealed that antimicrobial resistance is increasing among hvKp strains, which is consisted with a previous study [20]. However, the conclusion requires further investigation at multi-centres with a larger cohort of individuals to be confirmed. Moreover, the results show that the ESBL-hvKp is highly associated with magA in the study. The genetic characteristics and outer genetic environment of the two genes need to be further studied by whole genomic sequencing.

With regard to virulence factors, various types of K-antigens have been reported by now [24, 31, 32]. The most important elements are K1 and K2, which frequently result in serious infection [33, 34]. In our study, K1 and K2 are significantly higher in hvKp group than cKp group. RmpA/RmpA2 and MagA responsible for hypermucovicosity phenotype was proposed as another virulent factor in addition to cps K1/K2 [19, 23, 35, 36]. Our results showed that rmpA, rmpA2 and magA were closely related to hvKp group. These results revealed that most of the virulence factors are highly associated with this new definition of hvKp in the elderly.

Previous studies showed that hmvKp are frequently cause of invasive severe infection [37] in young people without underlying disease, such as PLA [2], suppurative endophthalmitis [38], and meningitis [39, 40]. In this study, the results show that the mean age of hvKp group is slightly younger than cKp. Invasive infection, especially liver abscess and other abscesses, occurred significantly more often with the new definition of hvKp group. In addition, the nutritional status (TP and ALB), host reaction (WBC and NEU %) and SOFA score of the hvKp group are significantly higher than cKp group. Moreover, the above results may also reveal that from the host, pathogen, and host–pathogen interactions, the new definition for hvKp may be highly associated with the real hypervirulence. Thus, focusing only on STs, serotypes, and other pathogen genomic data may not be sufficient to define hvKp. Host, pathogen and host–pathogen interactions should be taken into consideration when defining hvKp. The inflammatory factors (such as interleukin, C-reactive protein, tumour necrosis factor) and nutritional status (prealbumin, thickness of subcutaneous fat) may be more comprehensively considered in future studies.

It is essential for clinicians to respond immediately to hvKp infections, which could cause serious infections and a more severe inflammatory reaction than cKp, especially in the elderly, children and immunocompromised patients. Thus, developing a better understanding of the risk factors for hvKp is urgent and essential. Our results demonstrate that patients with diabetes and digestive diseases are more likely to be infected with hvKp, which is consistent with a previous study in China [14, 20]. Additionally, infections in the ICU and patients with indwelling stomach tube are risk factors for ESBL-hvKp, which may be related with potentially prolonged hospitalized course and antibiotic exposure. Clinicians should pay close attention to these risk factors in clinical practice to reduce emergence of MDR isolates. Previous study [28] suggested that wards previously infected with CR-hvKp should be left unoccupied for more than 2 weeks after disinfection and before the admission of new patients. However, it may be difficult to be implemented in China, a populous and developing country. Thus, it is urgent to make a cluster strategy from the host nutritional status, pathogen invasiveness and host–pathogen reaction to prevent MDR-hvKp, especially CR-hvKp.

There were some limitations in our study. First, it was a retrospective study at a single centre over 6 years. More inflammatory factors and nutrition indicators were not measured. Second, in vitro and in vivo experiments, such as galleria mellonella model, mouse models and a human neutrophil assay, may be further needed for identifying this new definition of hvKp. Third, to further explore the pathogen genomic characteristics, whole genome sequencing may be needed for further study. A prospective multi-centre study that includes more isolates, focusing on host, pathogen and host–pathogen interactions, is needed to better define the hvKp strains.

## Conclusions

The hvKp strains defined as hypermucoviscous and aerobactin positive are more likely to cause more severe inflammatory reaction in host and invasive infection, such as PLA and sepsis shock. To further understand hvKp, the host, pathogen and host–pathogen interactions may be the key element. At present, the prevalence of hvKp in the elderly, especially ESBL-hvKp and MDR-hvKp is increasing. It is essential to enhance the clinical awareness and management of hvKp infections.

## Additional file

**Additional file 1: Table S1.** Primers. **Table S2.** Comparison of antimicrobial resistance to hvKp and cKp.

## References

[1] Gupta A (2002). Hospital-acquired infections in the neonatal intensive care unit-*Klebsiella pneumoniae*. Semin Perinatol.

[2] Ko WC, Paterson DL, Sagnimeni AJ, Hansen DS, Von Gottberg A, Mohapatra S (2002). Community-acquired *Klebsiella pneumoniae* bacteremia: global differences in clinical patterns. Emerg Infect Dis.

[3] Podschun R, Ullmann U (1998). *Klebsiella* spp. as nosocomial pathogens: epidemiology, taxonomy, typing methods, and pathogenicity factors. Clin Microbiol Rev..

[4] Casanova C, Lorente JA, Carrillo F, Perez-Rodriguez E, Nunez N (1989). *Klebsiella pneumoniae* liver abscess associated with septic endophthalmitis. Arch Intern Med.

[5] Shon AS, Bajwa RP, Russo TA (2013). Hypervirulent (hypermucoviscous) *Klebsiella pneumoniae*: a new and dangerous breed. Virulence.

[6] Fang CT, Lai SY, Yi WC, Hsueh PR, Liu KL, Chang SC (2007). *Klebsiella pneumoniae* genotype K1: an emerging pathogen that causes septic ocular or central nervous system complications from pyogenic liver abscess. Clin Infect Dis.

[7] Decre D, Verdet C, Emirian A, Le Gourrierec T, Petit JC, Offenstadt G (2011). Emerging severe and fatal infections due to *Klebsiella pneumoniae* in two university hospitals in France. J Clin Microbiol.

[8] Pomakova DK, Hsiao CB, Beanan JM, Olson R, MacDonald U, Keynan Y (2012). Clinical and phenotypic differences between classic and hypervirulent *Klebsiella pneumonia*: an emerging and under-recognized pathogenic variant. Eur J Clin Microbiol Infect Dis.

[9] Yu WL, Fung CP, Ko WC, Cheng KC, Lee CC, Chuang YC (2007). Polymerase chain reaction analysis for detecting capsule serotypes K1 and K2 of *Klebsiella pneumoniae* causing abscesses of the liver and other sites. J Infect Dis..

[10] Wang JH, Liu YC, Lee SS, Yen MY, Chen YS, Wang JH (1998). Primary liver abscess due to *Klebsiella pneumoniae* in Taiwan. Clin Infect Dis.

[11] Catalan-Najera JC, Garza-Ramos U, Barrios-Camacho H (2017). Hypervirulence and hypermucoviscosity: two different but complementary *Klebsiella* spp. phenotypes?. Virulence..

[12] Zhang Y, Zeng J, Liu W, Zhao F, Hu Z, Zhao C (2015). Emergence of a hypervirulent carbapenem-resistant *Klebsiella pneumoniae* isolate from clinical infections in China. J Infect.

[13] Lin YC, Lu MC, Tang HL, Liu HC, Chen CH, Liu KS (2011). Assessment of hypermucoviscosity as a virulence factor for experimental *Klebsiella pneumoniae* infections: comparative virulence analysis with hypermucoviscosity-negative strain. BMC Microbiol.

[14] Zhang Y, Zhao C, Wang Q, Wang X, Chen H, Li H (2016). High prevalence of hypervirulent *Klebsiella pneumoniae* infection in china: geographic distribution, clinical characteristics, and antimicrobial resistance. Antimicrob Agents Chemother.

[15] Russo TA, Olson R, MacDonald U, Beanan J, Davidson BA (2015). Aerobactin, but not yersiniabactin, salmochelin, or enterobactin, enables the growth/survival of hypervirulent (hypermucoviscous) *Klebsiella pneumoniae* ex vivo and in vivo. Infect Immun.

[16] Russo TA, Olson R, Macdonald U, Metzger D, Maltese LM, Drake EJ (2014). Aerobactin mediates virulence and accounts for increased siderophore production under iron-limiting conditions by hypervirulent (hypermucoviscous) *Klebsiella pneumoniae*. Infect Immun.

[17] Gu D, Dong N, Zheng Z, Lin D, Huang M, Wang L (2018). A fatal outbreak of ST11 carbapenem-resistant hypervirulent *Klebsiella pneumoniae* in a Chinese hospital: a molecular epidemiological study. Lancet Infect Dis..

[18] Struve C, Roe CC, Stegger M, Stahlhut SG, Hansen DS, Engelthaler DM (2015). Mapping the evolution of hypervirulent *Klebsiella pneumoniae*. MBio.

[19] Siu LK, Yeh KM, Lin JC, Fung CP, Chang FY (2012). *Klebsiella pneumoniae* liver abscess: a new invasive syndrome. Lancet Infect Dis.

[20] Li W, Sun G, Yu Y, Li N, Chen M, Jin R (2014). Increasing occurrence of antimicrobial-resistant hypervirulent (hypermucoviscous) *Klebsiella pneumoniae* isolates in China. Clin Infect Dis.

[21] Magiorakos AP, Srinivasan A, Carey RB, Carmeli Y, Falagas ME, Giske CG (2012). Multidrug-resistant, extensively drug-resistant and pandrug-resistant bacteria: an international expert proposal for interim standard definitions for acquired resistance. Clin Microbiol Infect.

[22] Choi MJ, Ko KS (2015). Loss of hypermucoviscosity and increased fitness cost in colistin-resistant *Klebsiella pneumoniae* sequence type 23 strains. Antimicrob Agents Chemother.

[23] Yu WL, Ko WC, Cheng KC, Lee HC, Ke DS, Lee CC (2006). Association between rmpA and magA genes and clinical syndromes caused by *Klebsiella pneumoniae* in Taiwan. Clin Infect Dis.

[24] Cheng NC, Yu YC, Tai HC, Hsueh PR, Chang SC, Lai SY (2012). Recent trend of necrotizing fasciitis in Taiwan: focus on monomicrobial *Klebsiella pneumoniae* necrotizing fasciitis. Clin Infect Dis.

[25] Jung SW, Chae HJ, Park YJ, Yu JK, Kim SY, Lee HK (2013). Microbiological and clinical characteristics of bacteraemia caused by the hypermucoviscosity phenotype of *Klebsiella pneumoniae* in Korea. Epidemiol Infect.

[26] Lin JC, Yeh KM, Chang FY (2007). The distant metastasis of pyogenic liver abscess caused by *Klebsiella pneumoniae* serotype K2 and the underlying disease of diabetes mellitus should be carefully interpreted. Clin Infect Dis..

[27] Lin WH, Wang MC, Tseng CC, Ko WC, Wu AB, Zheng PX (2010). Clinical and microbiological characteristics of *Klebsiella pneumoniae* isolates causing community-acquired urinary tract infections. Infection.

[28] Zhang R, Lin D, Chan EW, Gu D, Chen GX, Chen S (2015). Emergence of carbapenem-resistant serotype K1 hypervirulent *Klebsiella pneumoniae* strains in China. Antimicrob Agents Chemother.

[29] Liu Y, Li XY, Wan LG, Jiang WY, Yang JH, Li FQ (2014). Virulence and transferability of resistance determinants in a novel *Klebsiella pneumoniae* sequence type 1137 in China. Microb Drug Resist.

[30] Siu LK, Huang DB, Chiang T (2014). Plasmid transferability of KPC into a virulent K2 serotype *Klebsiella pneumoniae*. BMC Infect Dis.

[31] Pan YJ, Fang HC, Yang HC, Lin TL, Hsieh PF, Tsai FC (2008). Capsular polysaccharide synthesis regions in *Klebsiella pneumoniae* serotype K57 and a new capsular serotype. J Clin Microbiol.

[32] Chuang YP, Fang CT, Lai SY, Chang SC, Wang JT (2006). Genetic determinants of capsular serotype K1 of *Klebsiella pneumoniae* causing primary pyogenic liver abscess. J Infect Dis.

[33] Yeh KM, Kurup A, Siu LK, Koh YL, Fung CP, Lin JC (2007). Capsular serotype K1 or K2, rather than magA and rmpA, is a major virulence determinant for *Klebsiella pneumoniae* liver abscess in Singapore and Taiwan. J Clin Microbiol.

[34] Brisse S, Fevre C, Passet V, Issenhuth-Jeanjean S, Tournebize R, Diancourt L (2009). Virulent clones of *Klebsiella pneumoniae*: identification and evolutionary scenario based on genomic and phenotypic characterization. PLoS ONE.

[35] Yeh KM, Chang FY, Fung CP, Lin JC, Siu LK (2006). magA is not a specific virulence gene for *Klebsiella pneumoniae* strains causing liver abscess but is part of the capsular polysaccharide gene cluster of *K. pneumoniae* serotype K1. J Med Microbiol.

[36] Fang CT, Chuang YP, Shun CT, Chang SC, Wang JT (2004). A novel virulence gene in *Klebsiella pneumoniae* strains causing primary liver abscess and septic metastatic complications. J Exp Med.

[37] Liu YM, Li BB, Zhang YY, Zhang W, Shen H, Li H (2014). Clinical and molecular characteristics of emerging hypervirulent *Klebsiella pneumoniae* bloodstream infections in mainland China. Antimicrob Agents Chemother.

[38] Abdul-Hamid A, Bailey SJ (2013). *Klebsiella pneumoniae* liver abscess and endophthalmitis. BMJ Case Rep..

[39] Tang LM, Chen ST, Hsu WC, Chen CM (1997). Klebsiella meningitis in Taiwan: an overview. Epidemiol Infect.

[40] Chang WN, Huang CR, Lu CH, Chien CC (2012). Adult *Klebsiella pneumoniae* meningitis in Taiwan: an overview. Acta Neurol Taiwan.

