# Supplementary material for: Hypervirulent Klebsiella pneumoniae (hypermucoviscous and aerobactin positive) infection over 6 years in the elderly in China: antimicrobial resistance patterns, molecular epidemiology and risk factor
Source: Ann Clin Microbiol Antimicrob. 2019 Jan 21;18:4. doi: 10.1186/s12941-018-0302-9 (PMC6341648; doi:10.1186/s12941-018-0302-9)
Supplement: Supplementary file 1 — Additional file 1: Table S1. Primers. Table S2. Comparison of antimicrobial resistance to hvKp and cKp. [file 12941_2018_302_MOESM1_ESM.docx]

Additional file 1: Table S1 Primers

| Name | Sequence |
| --- | --- |
| *rmpA* |  |
| Forward | 5-ACTGGGCTACCTCTGCTTCA-3 |
| Reverse | 5-CTTGCATGAGCCATCTTTCA-3 |
| *rmpA2* |  |
| Forward | 5-CTTTATGTGCAATAAG-GATGTT-3 |
| Reverse | 5-CCTCCTGGAGAGTAAGCATT-3 |
| *magA* |  |
| Forward | 5-GGTGCTCTTTACATCATTGC-3 |
| Reverse | 5-GCAATGGCCATTTGCGTTAG-3 |
| *aerobactin* |  |
| Forward | 5-GCATAGGCGGATACGAACAT-3 |
| Reverse | 5-CACAGGGCAATTGCTTACCT-3 |
| *K1* |  |
| Forward | 5-GTAGGTATTGCAAGCCATGC-3 |
| Reverse | 5-GCCCAGGTTAATGAATCCGT-3 |
| *K2* |  |
| Forward | 5-GGAGCCATTTGAATTCGGTG-3 |
| Reverse | 5-TCCCTAGCACTGGCTTAAGT-3 |
| *K5* |  |
| Forward | 5-GCCACCTCTAAGCATATAGC-3 |
| Reverse | 5-CGCACCAGTAATTCCAACAG-3 |
| *K20* |  |
| Forward | 5-CCGATTCGGTCAACTAGCTT-3 |
| Reverse | 5-GCACCTCTATGAACTTTCAG-3 |
| *K54* |  |
| Forward | 5-CATTAGCTCAGTGGTTGGCT-3 |
| Reverse | 5-GCTTGACAAACACCATAGCAG-3 |
| *K57* |  |
| Forward | 5-CGACAAATCTCTCCTGACGA-3 |
| Reverse | 5-CGCGACAAACATAACACTCG-3 |

Additional file 1: Table S2 Comparison of antimicrobial resistance to hvKp and cKp.

| Antibiotic agent | HvKp（80） | cKp（95） | P value |
| --- | --- | --- | --- |
| **ESBLs**  **Amikacin**  **Gentamicin**  **Ampicillin/Sulbactam**  **Aztreonam**  **Cefazolin**  **Cefepime**  **Ceftriaxone**  **Ceftazidime**  **Ciprofloxacin**  **Levofloxacin**  **Trimethoprim/Sulfamethoxazole**  **Piperacillin/ Tazobactam**  Imipenem  Meropenem  ampicillin  **Tobramycin** | **13 (16.3%)**  **2 (2.5%)**  **7 (8.8%)**  **16 (20.0%)**  **7 (8.8%)**  **15 (18.8%)**  **4 (5.0%)**  **13 (16.3%)**  **6 (7.5%)**  **8 (10.0%)**  **5 (6.3%)**  **7 (8.8%)**  **3 (3.8%)**  1 (1.3%)  2 (2.5%)  80 (100.0%)  **7 (8.8%)** | **38 (40.0%)**  **11 (11.6%)**  **28 (29.5%)**  **42 (44.2%)**  **22 (23.2%)**  **42 (44.2%)**  **14 (14.7%)**  **33 (34.7%)**  **24 (25.3%)**  **24 (25.3%)**  **21 (22.1%)**  **33 (34.7%)**  **13 (13.7%)**  2 (2.1%)  2 (2.1%)  93 (97.9%)  **26 (27.4%)** | **0.001**  **0.023**  **0.001**  **0.001**  **0.011**  **0.000**  **0.035**  **0.006**  **0.002**  **0.009**  **0.003**  **0.000**  **0.023**  0.665  0.862  0.501  **0.020** |
